# Multiscale perspectives of virus entry via endocytosis

**DOI:** 10.1186/1743-422X-10-177

**Published:** 2013-06-05

**Authors:** Eric Barrow, Anthony V Nicola, Jin Liu

**Affiliations:** 1 School of Mechanical and Materials Engineering, Washington State University, Pullman, WA 99164, USA; 2 Department of Veterinary Microbiology and Pathology, Washington State University, Pullman, WA 99164, USA; 3 Paul G. Allen School for Global Animal Health, Washington State University, Pullman, WA 99164, USA

**Keywords:** Viral entry, Theoretical modeling, Endocytosis, Multiple scales, Virus trafficking, Multiscale modeling

## Abstract

Most viruses take advantage of endocytic pathways to gain entry into host cells and initiate infections. Understanding of virus entry via endocytosis is critically important for the design of antiviral strategies. Virus entry via endocytosis is a complex process involving hundreds of cellular proteins. The entire process is dictated by events occurring at multiple time and length scales. In this review, we discuss and evaluate the available means to investigate virus endocytic entry, from both experimental and theoretical/numerical modeling fronts, and highlight the importance of multiscale features. The complexity of the process requires investigations at a systems biology level, which involves the combination of different experimental approaches, the collaboration of experimentalists and theorists across different disciplines, and the development of novel multiscale models.

## Introduction

Viruses are small but masterful infectious agents infecting all types of organisms from bacteria, plants and animals to humans. Emerging and re-emerging infectious diseases caused by viruses have taken a tremendous toll on human health worldwide [1-3]. For instance, human immunodeficiency virus (HIV) has killed more than 25 million people since 1981. ~ 3% of the world’s population has been infected with hepatitis C virus, which causes 350,000 deaths annually. Influenza A virus infection results in hundreds of thousands of deaths each year and has caused some of the deadliest pandemics in human history.

Viruses are obligatory intracellular parasites and therefore must enter host cells and deliver their genetic material to initiate infection. The plasma membrane of a host cell represents the first physical barrier that viruses must overcome to gain entry. Some enveloped viruses such as herpes simplex virus type 1 (HSV-1), Sendai virus and HIV are able to penetrate into cells by direct fusion with the plasma membrane. However, the majority of viruses take endocytic pathways to enter host cells for productive infection. For recent reviews see [4-8]. Endocytosis is the process by which an intracellular vesicle is formed by membrane invagination resulting in engulfment of extracellular and membrane-bound components [9,10]. Endocytosis plays a central role in control of essential cellular functions, modulation of membrane composition and regulation of many intracellular signaling cascades. Since endocytosis can transport relatively large particles, it is therefore critically important for the design of nanomedicines and targeted drug delivery [11]. Viruses entering cells via endocytosis offers many advantages [8]. For instance, endocytosis allows animal viruses to bypass obstacles posed by plasma membrane and cytoplasmic crowding, and by the meshwork of microfilaments in the actin cortex. It can also allow direct transport to subcellular sites of viral replication. Furthermore, endocytosed viruses can overcome host immune surveillance by leaving minimal evidence on the cell surface. Indeed, it has been suggested that HIV entry by endocytosis is favored over direct fusion with the plasma membrane [12].

Viruses take advantage of a number of endocytic mechanisms for entry. In clathrin-mediated endocytosis (CME), clathrin-coated vesicles (CCVs) are formed by the nucleation of clathrin-coated pits (CCPs) induced by receptors and bound ligands [10]. CME is the main pathway for internalization of extracellular components; therefore it is the most well characterized and extensively studied endocytic pathway. Macropinocytosis is a transient, growth factor induced and actin-dependent process, which often involves the formation of membrane ruffling and large vacuoles [13]. This mechanism is usually used for internalization of fluid and membrane, but it can also drive the uptake of viruses [14-16]. Several nonenveloped viruses, such as simian virus 40 (SV40) [17] and polyoma virus [18], enter host cells through caveolin-mediated endocytosis, in which caveolae composed of a coat of caveolin proteins are formed [19]. Other novel endocytic pathways, which are clathrin and caveolin independent, have also been identified [20-22] but remain poorly characterized due to the relative lack of known marker proteins. Depending on the virus, cell type, and local microenvironment variables (such as local pH), viruses are able to exploit a number of different endocytic pathways to gain entry.

**Figure 1:**
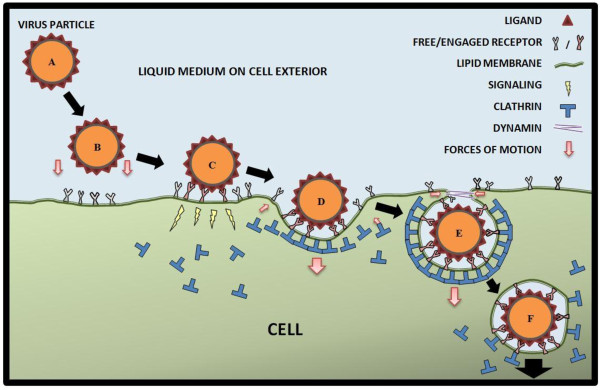
**Illustration of the steps of virus entry via clathrin-mediated endocytosis.** (**A**) Virus approaches the cell surface. (**B**) Biochemical interactions between ligands and receptors attract virus to the cell surface. (**C**) Virus attaches to the cell surface and signals the cell. (**D**) A clathrin-coated pit is formed around the bound virus. (**E**) A clathrin-coated vesicle is formed, and the dynamin at the neck region facilitate vesicle scission. (**F**) The vesicle travels to cell interior.

Despite much progress, a systematic and mechanistic understanding of viral entry into host cells through endocytosis is still elusive. As illustrated in Figure 1 in terms of CME, this is a complex process, which involves viral ligands and likely hundreds of cellular proteins, such as virus receptors, and the multifactorial machineries and signaling pathways associated with the clathrin, actin, dynamin, caveolin, and microtubule networks to name a few [23]. The entire process is dictated by the collective and cooperative interplay of various events, such as virus motions, membrane deformations, receptor diffusion and ligand-receptor interactions, occurring at multiple length and time scales. Due to the multiscale nature of the problem, it is extremely difficult to explore the whole process using a single technique. Viral endocytosis and related topics have been previously reviewed and discussed [6,8,13,24-27] with focuses on different aspects. Here, we review recent technical advances in viral endocytic entry with an emphasis on the multiscale features of the process, and the collaborative and complementary roles played by experimentation and theoretical/numerical modeling. Section 2 describes the different experimental techniques utilized in different scales to explore viral endocytic entry. In section 3, we review progress on the modeling and simulation fronts. Conclusions and future perspectives are given in section 4.

## Experimental techniques

Exploring viral endocytic entry mechanisms is challenging because of the multiple, diverse entry pathways and the multiple stages therein. Each pathway involves numerous physical and chemical interactions between the virus and host cells. Recent advances in experimental techniques at different scales have improved our understanding of this important process.

### Cellular scale information: electron microscopy and fluorescence microscopy

**Figure 2:**
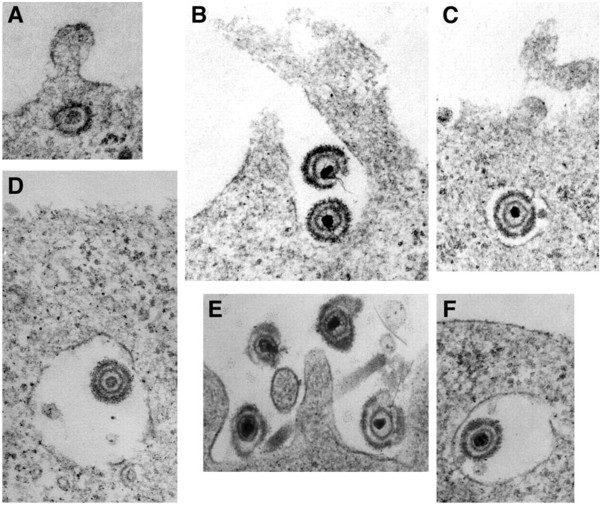
**Electron microscopic analysis of HSV-1 uptake into cells.** HSV-1 was bound to Vero (**A**), CHO-nectin-1 (**B** to **D**), or HeLa (**E** and **F**) cells for 2 h at 4**°**C. This was followed by a shift to 37**°**C for 20 min, and then the cells were processed for electron microscopy. Reproduced with permission from reference [29].

Electron microscopy (EM), including scanning electron microscopy (SEM) and transmission electron microscopy (TEM), can provide still images with resolution close to the nanometer scale. For more than 50 years, EM has been routinely exploited in research on virus entry to provide structural and compositional information [28]. As a more recent example, Figure 2 shows electron micrography of HSV entry via either endocytosis or by direct penetration at the plasma membrane depending on the cell type [29]. When combined with functional studies that measure successful entry of particles that initiate productive infection, EM analysis has supported the notion that both endocytic and non-endocytic pathways are productive for HSV entry.

However, EM experiments are usually performed under restricted conditions and on fixed cells, which is often incompatible with a live-cell environment. Furthermore, important dynamic interactions are usually overlooked in EM measurements. On the other hand, live-cell microscopy experiments are able to provide more dynamic information. Fluorescent-labeling of viruses and cellular structures combined with fluorescence microscopy has allowed for tracking of single virus in live cells and direct visualization and quantitative analysis of cellular dynamics [30]. Single viral particle-tracking revealed the caveolar endocytic entry of SV40 [31]. By tracking the dynamics of single influenza viruses and their interactions with cellular endocytic structures, Rust *et al*. [22] showed that influenza can enter cells using both clathrin-mediated and clathrin/caveolin-independent endocytic pathways with comparable efficiency. In addition, a virus-induced CCP formation mechanism was suggested by tracking the interaction between viruses and CCPs in real time. Single particle analysis combined with the use of a dominant-negative mutant identified epsin 1, a clathrin-interacting protein, as a cargo-specific adaptor during the endocytic entry of influenza virus [32]. Using a similar combination of methodologies, Dengue virus was found to enter live cells exclusively through CME [33]. Single particle tracking demonstrated distinct motions of murine polyoma virus-like particles [34] and human papillomavirus 16 [35] on the cell surface. Moreover, it was also found that poliovirus entered the cell by a clathrin, caveolin, flotillin and microtubule-independent, but tyrosine kinase and actin-dependent endocytic pathway [36,37].

Although single-virus analysis has revealed previously undetectable events in viral entry, the spatial resolution of conventional optical microscopy remains limited to a few hundred nanometers by the diffraction effect of light. However, virus-cell molecular interactions occur at nanometer scales. Several promising super-resolution optical microscopy techniques have been developed [38-41] and these techniques have pushed the spatial resolution to nanometer scales. Most recently one of the techniques, stochastic optical reconstruction microscopy (STORM), has been successfully utilized to quantify the size of the HIV-1 matrix shell and capsid core with a lateral resolution of 15–20 nm [42]. The results agreed with HIV-1 organization seen by EM.

### Electron tomography

Besides physical and biochemical interactions, the geometrical parameters (such as the shape and size) of the viral particles, as well as the structural and distributional information of the envelope glycoprotein complexes also play vital roles in virus endocytic entry. For example, influenza A has pleiomorphic virions. Spherical virions utilize CME to enter host cells; however, filamentous virions use macropinocytosis as the primary entry mechanism [43].

Electron tomography (ET), an extension of traditional EM, is a technique to visualize and characterize the three-dimensional (3D) structures of subcellular macromolecular objects by reconstruction of a series of projected images taken at different angles of the target. ET is particularly powerful and advantageous in obtaining 3D geometrical and distributional information for non-symmetric heterogeneous objects in native form with resolution in the nano and subnanometer scales (atomic scales).

The architectures of various viral particles, including the shapes of the virions and the envelope glycoprotein complexes, have been actively explored since the emergence of the ET technique [44]. For example, by visualizing isolated HSV by cryo-ET, a combination of cryo-microscopy and electron tomography, the glycoproteins on the HSV envelope were revealed to be distributed in a nonrandom fashion, varying in length, spacing and angles [45]. In reference [46], an iterative 3D averaging algorithm was developed and utilized with cryo-ET measurements to determine the structure of the envelope glycoprotein complex of Moloney murine leukemia virus (MoMuLV) with a resolution of 2.7 nm. Other types of viruses, including vaccinia virus [47], human and simian immunodeficiency viruses (HIV and SIV) [48,49], influenza virus [50] and Ebola virus [51], have also been investigated using cryo-ET techniques.

**Figure 3:**
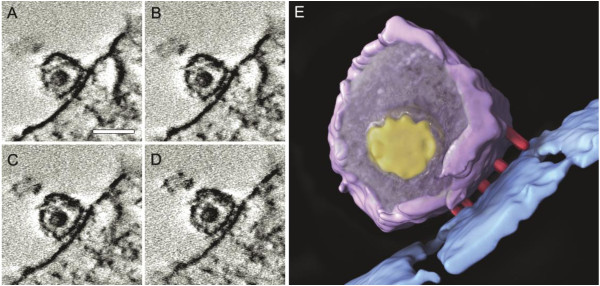
**Imaging of HIV-1 in contact with T cells.** (**A**-**D**) Four slices at different depths in a tomogram of the contact between HIV and T cells. (**E**) 3-D tomographically derived architecture of the contact region. Scale bar in (**A**) is 100 nm. Reproduced with permission from reference [53].

The architectures, including the interacting ligand-receptor pairs and deforming cellular membrane, surrounding the contact zone between the virus and host cell during entry have significant implications in understanding mechanisms of virus endocytic entry. ET provides an ideal tool to visualize and analyze virus-cell interactions. Recently, ET has been employed to study different stages of the virus life circle [52]. Figure 3 (E) shows a tomographical image of HIV-1 in contact with T cells [53] which is reconstructed from EM images (A-D). As shown, a neck-shaped contact zone, which is named an “entry claw”, is identified. This contact zone is ~40 nm wide and is composed of a number of closely spaced rod-shaped structures. However, when anti-CD4 antibodies, the CCR5 antagonist TAK779, or the peptide entry inhibitor C34 are introduced, the contact zone cannot be observed. By analyzing a series of ET images at different stages of HSV-1 [54] or gammaherpesvirus [55] infection, molecular details of the structural changes including membrane deformation are revealed during the entry processes. Similarly, the interactions between vaccinia virus [56], filovirus [57] and their respective host cells have been studied using ET techniques.

### Molecular scale interactions: single-molecule force spectroscopy

Virus entry pathways are primarily determined by the interactions between virus particles and the corresponding cellular receptors [58]. The interactions between viral ligands and cellular receptors are specific and dynamic, and involve molecular scale conformational changes and signaling events. Experimental measurement of the interactions at a single molecule scale is challenging but vital to understanding viral endocytic entry.

One method of quantifying ligand-receptor interactions is the optical biosensor assay, in which the kinetic rate constants are determined by analysis of surface plasmon resonance of a glass surface before and after ligand binding. Due to its ease of use and the high throughput nature of the experimental setup, this method has been widely used to characterize the specific interactions between viral ligands and cellular receptors. For instance, in references [59,60] the kinetic rate constants (*k*_*on*_ and *k*_*off*_) for HSV glycoprotein D binding to its cellular receptors are measured using optical biosensor assays. However, due to the inherent limitations of the controlled environment, certain dynamic information about virus entry (e.g., conformational changes and signaling events) cannot be captured by this technique.

On the other hand, atomic force microscopy (AFM) provides a powerful and versatile tool to study molecular scale interactions. In AFM, a very tiny tip mounted at the end of a microcantilever is controlled to probe a sample. The deflection of the cantilever is recorded and translated to topographical or mechanical information. Recently, AFM has received much attention in the field of biophysics [61]. In bio-imaging applications, AFM is implemented to investigate the distribution and interaction of lipids and proteins on lipid bilayers at the single molecule level [62]. In bio-force measurement applications, the elastic properties of the lipid bilayer, the bending modulus, the lateral tension and the adhesion constant, can be simultaneously determined by using AFM force measurement on a lipid membrane surface [63]. AFM is also applied to probe the differences in mechanical properties between normal and cancer cells [64,65]. Physical and mechanical insights from AFM measurements may be important in understanding cancer metastasis [66].

**Figure 4:**
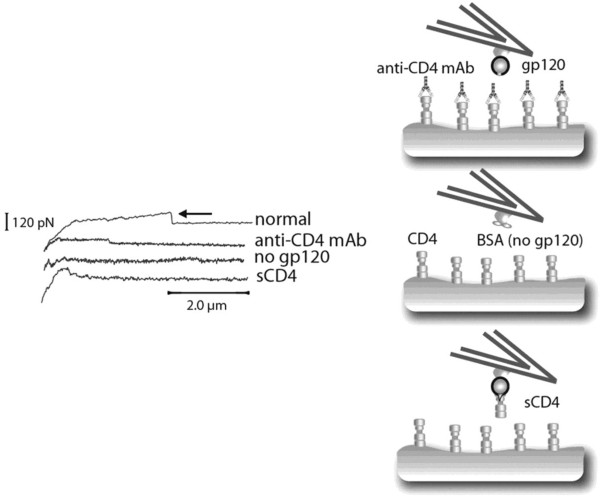
**The interactions between gp120-coated cantilevers and cell receptors are specific.** Reproduced with permission from reference [69].

The initial interaction of HIV with the host cell is the binding of the viral surface ligand gp120 to cell surface CD4 molecules [67,68]. Wirtz and co-workers have implemented AFM to analyze the interactions between HIV-1 envelope glycoprotein and corresponding receptors [69]. In their experiments, the glycoprotein gp120 is attached to the cantilever tip. The tip is controlled to probe a living cell containing the receptor CD4 and/or the coreceptor CCR5. Figure 4 (left) illustrates the force-distance curves recorded by AFM measurements of interactions between gp120 and CD4 under different conditions. As shown, a rupture event indicating binding interactions can be observed under normal conditions. However, no binding can be detected if the CD4 is blocked by an anti-CD4 function-blocking monoclonal antibody (mAb) (right-top), the gp120 is absent (right-middle), or the gp120 is blocked by sCD4. Furthermore, by measuring the binding strength of gp120-CD4 with CCR5 and comparing with gp120-CD4 without CCR5, the dynamics and mechanisms of the receptor/coreceptor mediated interactions, and their implications on HIV entry are discussed. In later work [70], the detailed tensile strength and lifetime of the gp120-CD4 bond are measured. A destabilization of the bond is enhanced by the coreceptor CCR5, and this is directly related to a conformational change in the gp120-CD4 bond.

## Theoretical and numerical models

The entire process of viral endocytic entry is dictated by dynamic interplay among various biomechanical and biochemical interactions occurring in multiple spatial and time scales. Significant advances in experimental techniques as mentioned above have provided indispensable data from different scales. Meanwhile, a number of theoretical and numerical models have been developed recently to further improve our fundamental understanding of the mechanisms of viral endocytic entry.

### Theoretical continuum models

Theoretical models have been developed to provide fundamental insights into viral endocytic entry into host cells. In such models, the total free energy functional of the system is formulated by considering both energetic and entropic contributions in endocytic events, such as receptor diffusions, ligand-receptor interactions and membrane deformations. Those energetic and entropic contributions are approximated by continuum functions. Then by considering the thermodynamic equilibrium or by minimizing the free energy functional, the effects of different parameters on viral endocytic entry can be explored.

Using a theoretical continuum model, van Effenterre and Roux [71] have discussed the effect of the volume concentration on viruses binding to a planar membrane. A relationship between the virus resting time on the cell surface and virus volume concentration was derived, based on which an optimal volume concentration for virus internalization was identified. Gao *et al.*[72] developed a model in the context of explaining the mechanism of clathrin-free viral endocytic entry into cells. By analyzing the wrapping time for a viral particle, the authors identified a threshold particle radius (~12 nm for cylindrical and ~24 nm for spherical particles) and a threshold receptor density, below which clathrin-independent endocytosis would not occur. By considering the competition between thermodynamic driving force and receptor diffusion kinetics, an optimal particle radius for endocytosis was derived (~15 nm for cylindrical and ~30 nm for spherical particles). Interestingly, the predicted results agreed remarkably well with experiments from Aoyama and colleagues [73-75]. Recently, the effect of elastic deformation of particles on cellular uptake was studied using a theoretical model [76]. The cellular internalization of particles was strongly dependent on the particle elasticity. Phase diagrams describing the transition between different wrapping phases were generated based on their analysis.

The continuum model developed by Sun and Wirtz [77] incorporated the energy contributions from the elastic deformations of the cell membrane and cytoskeleton, and the ligand-receptor interactions. From their analysis, the energy due to the deformation of cytoskeleton was much greater than the other energy contributions and this led to the conclusion that cytoskeleton deformation is the dominant determinant of endocytosis. However, as pointed out recently by Li *et al.*[78], the analysis in reference [77] underestimated the contribution from membrane deformation, and virus internalization should be dictated by the membrane deformation once the viral particle is beyond a critical size, which is dependent on the Young’s modulus of cytoskeleton. Recent work by Decuzzi and Ferrari has considered the effects from the non-specific interactions [79] and particle nonsphericity [80] on receptor-mediated endocytosis (RME) for cylindrical particles. It was shown from their analysis that the contribution from non-specific interactions could be as important as those from specific ligand-receptor interactions; the particle shape and size also played critical roles in RME.

Recently, Zhang and coworkers [81] presented a theoretical analysis of the particle size effect on the number of nanoparticles (or virions) that can be internalized into the cell. Interestingly the optimal particle size for maximal cellular internalization predicted in their model agreed well with the predictions in reference [72]. Later the effects of particle size and ligand density on RME were discussed [82,83]. By analyzing the free energy functional, it was found that there existed a minimal particle size at a given ligand density and a minimal ligand density at a given particle size, below which endocytosis is not possible. The internalization rate interrelatedly depends on the particle size and ligand density, and an optimal condition (25–30 nm in particle radius and several tens of coated ligands) exists at which the endocytic rate is maximal. This finding is consistent with the structure of Semliki Forest virus, which has a radius of ~ 35 nm and is covered with 80 glycoproteins.

### Stochastic mesoscale models

A number of stochastic mesoscale models have been developed to study virus binding to host cell surfaces. In these models, the viral particles are treated as rigid spheres whose surfaces are decorated with ligand proteins. The viral particles bind to the cell surface through interactions between ligands and receptors on the cell surface. Both ligand and receptor proteins are coarse-grained, and the interaction potentials are determined from independent biophysical experiments. By accounting for the total force acting on the viral particles, the trajectories of viral particles can be calculated by solving the equations of motion.

English and Hammer [84] implemented Brownian Adhesive Dynamics (BRAD) to simulate the receptor-mediated binding of viruses. In their model, the parameters were chosen to mimic the interactions between HIV particles and host cell surfaces. The cell membrane was treated as a rigid surface, and the kinetic rates of gp120-CD4 binding were used as the ligand-receptor interactions. The binding of HIV particles to CD4-expressing cells were simulated using BRAD, and the results were compared with results predicted by an equivalent site hypothesis. Dramatic differences in the transition rates were found. In later contributions, CD4 receptor proteins were allowed to diffuse freely in the plane of the membrane [85], and the simple bead-spring model was developed to coarsely approximate the structure of glycoprotein gp120 [86]. Then the roles of cellular receptor diffusion and gp120 trimerization on HIV binding were investigated. The important finding was that it took seconds for gp120 to cluster CD4 in the contact zone, and this might partially explain the delay in viral entry. Using similar modeling strategies, Dobrowsky *et al.*[87] developed a stochastic model to investigate the spatial and temporal organization of cellular receptor CD4 and co-receptor CCR5 at the plasma membrane during HIV adhesion. In their model, the deformation of plasma membrane was evaluated by displacement of discrete points in Cartesian coordinates. CD4 and CCR5 were allowed to move on the membrane, and they were able to bind to gp120 on the HIV surface. The kinetic and micromechanical parameters for gp120-CD4 binding were obtained from direct experimental measurements [70]. A well-organized, ring-like, nanoscale structure beneath the virion, which the authors named the viral junction, was observed as a result of binding of gp120 to CD4 and CCR5. The local plasma membrane deformed in response to the formation of the viral junction. Similar structures were also reported in a tomographical image of HIV-1 in contact with T cells [53]. The authors speculated that the formation of the nanoscale viral junction might well correlate with the complex signaling events during viral entry, such as the assembly of a local actin network and the disassembly of actin filaments.

**Figure 5:**
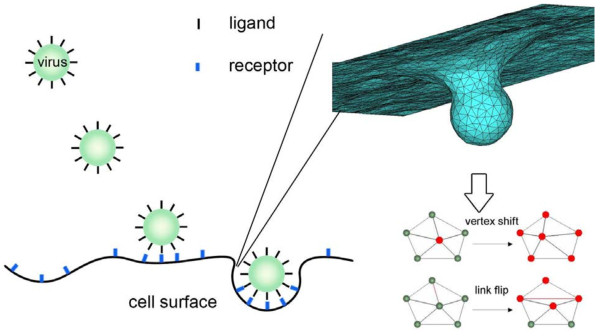
**Schematic of the mesoscale model for virus endocytic entry.** The virus is modeled as a sphere decorated with ligands. The cell surface is modeled as a plasma membrane with diffusive receptors. The membrane surface is discretized by a curvilinear triangulate system.

In drug delivery of functionalized nanocarriers (NCs) to endothelial cell (EC) surfaces, similar to RME of viral particles, NCs first bind to cell surface via ligand-receptor interaction and then enter the cell through RME. Recently, Liu *et al.*[88] developed a mesoscale binding model to study the drug delivery to EC surfaces using NCs. Briefly, in their model both the ligands on the NC surface (anti-ICAM1) and receptors on the EC surface (ICAM1) are coarse-grained as cylinders. The ICAM1s are allowed to diffuse on the EC surface, and the interactions and physical/mechanical parameters are determined from independent experiments. Using this model the authors developed a methodology to quantify the binding free energy or potential of mean force (PMF) between the NC and EC surfaces. Then the binding affinities were calculated based on the PMF profiles. The authors implemented this model to study the effect of anti-ICAM1 surface coverage of NC on binding and revealed a threshold value, below which the NC binding affinities decrease drastically and drop lower than that of a single anti-ICAM1 molecule to ICAM1. Interestingly, this prediction agrees remarkably well with experimental results of *in vivo* targeting of anti-ICAM1 coated NCs to pulmonary endothelium in mice. By analyzing the simulation results, it was revealed that the dominant effect of changing antibody surface coverage around the threshold is through a change in multivalent interactions. Moreover, the model results of NC rupture force distribution agree well with corresponding AFM experiments. The model was further extended to investigate effects of particle size, shear flow and resistance due to the existence of glycocalyx [89,90]. Intriguingly, all the model predictions agreed with the corresponding experiments. The mesoscale model developed in the context of drug delivery can be readily applied to study the binding of viral particles. A significant drawback in the above models is that the host cell membrane is either treated as a rigid surface or as a surface with small deformations. This restricts the discussions to the early adhesion of viral particles. A more flexible membrane model that can accommodate extreme deformations has been discussed in references [91,92], and is needed for these mesoscale models to analyze viral endocytic entry (see Figure 5 for illustration).

### Discrete models

Full detailed molecular dynamics (MD) simulations are able to provide three-dimensional real-time information of the system with the finest atomistic level resolution. In principle, this can resolve all the structural and dynamic details. However, MD simulations are time consuming and are restricted to exploring systems with small spatial and temporal scales. For example, it will be difficult to simulate a lipid bilayer system consisting of more than hundreds of hydrated lipids for micron seconds using full detailed MD under current computational resources. Considering the spatial and temporal scales involved in viral endocytic entry, it is impractical to simulate using full detailed MD. Recently, a number of coarse-grained MD [93,94] and dissipative particle dynamics (DPD) [95-98] simulations have been performed to explore the process of RME of nanoparticles (NPs). In such models, the lipid, ligand and receptor molecules are represented by a number of beads connected to each other. Each bead approximates the effect of many molecular atoms. The force on each bead and therefore the trajectory can be calculated through interaction potentials among different beads. In DPD, three types of forces, namely conservative, dissipative and random forces, are considered. The RME of NPs can be modeled by adjusting the interaction parameters. Through such coarse-graining techniques, the simulations can be extended to much larger spatial and temporal scales while retaining a certain degree of discrete information.

Yue and Zhang [95] presented a study on the receptor-mediated membrane responses to a ligand-coated NP using DPD simulations. Four types of membrane responses were observed in simulations: membrane rupture, NP adhesion, NP penetration and RME. The effects of NP size, membrane tension and ligand density on membrane response were discussed and phase diagrams were generated based on discussions. The effects of particle shape anisotropy on RME were studied in a later contribution [96]. Most recently the authors also investigated the pathways of the interaction between elastic vesicles and lipid membranes [98]. Using similar DPD simulations, Ding and Ma [97] have discussed the RME of NPs focusing on the effect of the coating ligand properties. Both the biochemical property (ligand-receptor interaction strength) and biophysical properties (length, rigidity and density) of the ligands are studied. Both biochemical and biophysical properties actively impact the efficiency of NP engulfment. Vacha *et al.*[93] have investigated the effects of size and shape of NPs on RME using coarse-grained MD simulations. Larger spherical particles entered the cell more readily than smaller ones due to a more favorable compromise between bending rigidity and surface adhesive energy. In addition, the spherocylindrical particles could be internalized more efficiently than spherical ones. Shi *et al.*[94] employed coarse-grained MD simulations to study the cell entry of carbon nanotubes. However, due to the computational cost, the sizes of the NPs (or vesicles) considered in these simulations are relatively small (~10 nm in diameter).

## Conclusions

Most viruses exploit endocytic pathways to enter cells to initiate infection. Thus a systematic and mechanistic understanding of the virus endocytic entry process is critically important for the development of targeted and specific inhibitors of virus entry and infection [99]. The events involved in the process are dynamic and span multiple spatial and temporal scales. Information about the process is accumulating with the advent of new and powerful experimental technologies as we have discussed in section 2. However, the experimental complexity of a single technique usually restricts observations to a specific spatial and temporal scale and therefore can only describe part of the whole story. Optical and electron microscopy are able to directly provide information on a cellular level but neglect molecular level events. Single-molecule force spectroscopy is able to probe interactions at the molecular level but overlooks the effects of the microenvironment. The question of how to interpret and integrate these seemingly scale-specific data and provide a systematic view of the whole process is a significant challenge.

From a modeling point of view, similar situations are present. Ideally we hope to model the whole viral endocytic entry process with resolution at the molecular scale. Theoretical continuum models (macroscale models) have the advantage of efficiency and are able to resolve cellular level events, but the molecular scale details cannot be resolved. On the other hand, discrete atomistic models (microscale models) are able to provide all the molecular details but are computationally prohibitive to simulating a realistic system. As a result, recently much effort has been devoted to the development of multiscale models. In such models, a microscale model is integrated into a macroscale model so that the expensive microscale calculations only take place in necessary places. By proper design of the communications across different models (scales), a multiscale model has the advantages of both the efficiency of the macroscale models and the accuracy of the microscale models. The biggest challenge in a multiscale model is how to handle the interfaces between different models where information should be mutually exchanged. Some methodologies have been developed, though, in other disciplines [100-104].

Finally, the complexity and multiscale nature of the viral endocytic entry process requires investigation at a systems biology level. This demands interdisciplinary knowledge and expertise in cellular, structural and molecular biology, as well as the collaborative and complementary work between experimental and theoretical/modeling research. Here we would like to emphasize the important role of collaboration between experimentalists and theorists. Theoretical and numerical models have the power of mechanistically interpreting data uncovered in experiments, and making predictions to guide further experimental design. However, all models must eventually be faithful to experiments and must be rigorously validated through experimentation before making predictions. The model parameters must be evaluated from the corresponding experiments. Furthermore, a model should be versatile and adaptive to modifications to incorporate new important factors discovered by experimentation. This process involves iterative validation and verification between modeling and experiments. With the emerging novel and more quantitative experimental technologies, as well as the new modeling methodologies, especially the multiscale modeling methods, a definitive and mechanistic understanding of viral endocytic entry is taking place.

## Competing interests

The authors declare they have no competing interests.

## Authors’ contributions

EB, AVN, and JL participated in the writing of the manuscript. All authors have read and approved the final manuscript.
